# Clinical, Biological, and Imaging Features of Monogenic Alzheimer's Disease

**DOI:** 10.1155/2013/689591

**Published:** 2013-11-27

**Authors:** Andrea Pilotto, Alessandro Padovani, Barbara Borroni

**Affiliations:** Clinica Neurologica, Università degli Studi di Brescia, Pza Spedali Civili, 1-25100 Brescia, Italy

## Abstract

The discovery of monogenic forms of Alzheimer's Disease (AD) associated with mutations within *PSEN1, PSEN2,* and *APP* genes is giving a big contribution in the understanding of the underpinning mechanisms of this complex disorder. Compared with sporadic form, the phenotype associated with monogenic cases is somewhat broader including behavioural disturbances, epilepsy, myoclonus, and focal presentations. Structural and functional imaging show typical early changes also in presymptomatic monogenic carriers. Amyloid imaging and CSF tau/A**β** ratio may be useful in the differential diagnosis with other neurodegenerative dementias, especially, in early onset cases. However, to date any specific biomarkers of different monogenic cases have been identified. Thus, in clinical practice, the early identification is often difficult, but the copresence of different elements could help in recognition. This review will focus on the clinical and instrumental markers useful for the very early identification of AD monogenic cases, pivotal in the development, and evaluation of disease-modifying therapy.

## 1. Introduction

Between 1991 and 1995 different families of early onset Alzheimer's Disease (EOAD) have been linked to mutations within Presenilin (*PSEN1, PSEN2*) and Amyloid Precursor Protein (*APP*) genes [1–3]. The discovery of monogenic forms of Alzheimer's Disease (AD) has allowed improved knowledge of the physiopathology which, in turn, has allowed the design of new therapeutic strategies. After 20 years of basic and clinical research, understanding the early phases of monogenic AD has become pivotal in order to develop and test the efficacy of the newest target-therapeutic approaches [4].

Even though most monogenic forms of AD have been described in familial early onset AD, recent findings suggest a wider spectrum of clinical presentation, including late-onset and sporadic forms. Indeed, monogenic AD might present a wide body of clinical symptoms beyond memory deficits, and the careful characterisation is key for a proper diagnosis in unclear cases.

The present review will focus on the clinical and instrumental markers that should be considered in the identification of different forms of monogenic AD.

## 2. Epidemiology

The incidence and distribution of different forms of early onset (or presenile, <65 years) dementia (EOD) are still the theme of controversy [5–7]. When considering neurodegenerative conditions, most of the studies showed AD as the most common aetiology in EOD [5, 7–10], although recent findings indicate that frontotemporal lobar degeneration (FTLD) may have a similar or even higher incidence at this age [11]. Also the relative contribution of *PSEN1*, *PSEN2*, and *APP* mutations to early onset Alzheimer's Disease (EOAD) is the subject of considerable controversy, and mutation frequency is highly dependent upon the studied population [12–15].

*PSEN1* mutations are considered as the major cause of familial AD, accounting for 18 to 55% of families ([12–14], http://www.molgen.ua.ac.be). The second most common monogenic form of Alzheimer's Disease involves *APP* mutations and duplication, accounting for 2–18% and 8% of autosomal dominant early onset cases, respectively [12, 14, 16–18]. *PSEN2 *mutations are rare, with only 22 families reported so far (http://www.molgen.ua.ac.be).

## 3. Genes and Pathophysiology of Monogenic AD

Monogenic AD shares neuropathology features with sporadic AD. Neuronal and synapse loss, extracellular plaques composed of amyloid-*β*(A*β*) peptides, and intraneuronal neurofibrillary tangles consisting of hyperphosphorylated tau protein [19] are the specific features of the disease [20]. All mutations in Presenilins and *APP* genes lead to increased amyloidogenic processing of APP, causing the deposition of A*β* peptide, the primary component of amyloid plaques deposition [19, 21]. The *APP* gene has 18 exons and encodes an alternatively spliced transcript that, in its longest isoform, expresses a single transmembrane spanning polypeptide of 770 amino acids that is subject to at least two independent proteolytic pathways. The bulk of APP is cleaved by *α*-secretase within the A*β*-domain to produce a C-terminal fragment, which can be further cleaved intramembranously by *γ*-secretase to produce the peptide P3 and the transcriptionally active APP intracellular domain [22]. Alternatively, APP can be sequentially cleaved to produce A*β* peptide, which requires initial cleavage of APP by *β*-secretase, followed by *γ*-secretase cleavage [23] within the single-transmembrane domain. If cleavage occurs at residue 712–713, the most common short A*β* (A*β*1-40) results; if it is after residue 714, the longer A*β*42 is generated [24]. A*β*1-42 has a higher propensity to form aggregates and has been associated with AD pathology as component of extracellular amyloid plaques [19, 25, 26]. Presenilins with nicastrin, aph-1, and pen2 are required for the stability and activity of the *γ*-secretase complex [27].

### 3.1. *APP* Mutations

Interestingly, most of *APP* mutations are located at the *γ*-secretase cleavage sites or the APP transmembrane domain on exons 16 and 17, influencing APP processing. The substitutions near the proteolytic sites lead to an overproduction of total amyloid-*β* or a shift in the A*β*1-40/A*β*1-42 ratio towards formation of the more toxic A*β*1-42 peptide. The substitutions within the APP transmembrane domain result in formation of amyloid-*β* with increased propensity for aggregation [26]. In addition to more frequent dominant *APP* mutations, two recessive mutations causing disease only in the homozygous state were identified: a trinucleotide deletion E693D segregating in one Japanese family proportionally decreased A*β*40 and A*β*42 with no change in their ratio [25] and A673V in one other Italian family [28]. Additionally, the mutation spectrum extended to *APP* locus duplications underscoring the importance of *APP* gene dosage in AD, already observed in the case of Down syndrome [29]. Duplicated *APP *regions containing several genes [16, 30] or *APP *only [17] have been clinically linked to early-onset AD often with extensive cerebral amyloid angiopathy [31].

The mutation A673T within *APP *was found to be protective against AD and age-related cognitive decline in a study in Iceland with the evidence of a 40% reduction in the formation of amyloidogenic peptides in vitro [32]. These findings are not completely understood, given the homozygous presence of the same A673Tsubstitution in a very early onset AD in a single Italian family [28]. On the other side, Jonnson and colleagues identified three homozygous carriers of A673T in Icelandic samples, one of whom had died at age of 88, whereas the other two were currently living at age of 67 and 83, respectively, and none had a history of dementia [32].

### 3.2. *PSEN1* and *PSEN2* Mutations

PSENs are functionally involved in the *γ*-secretase-mediated proteolytic cleavage of APP [21]. Thus, mutations in PSENs result in an increased A*β*42/A*β*40 ratio, by either an increase in A*β*42 as shown in plasma and fibroblast media of PSEN mutation carriers [33] or by a decrease in A*β*40, suggesting a loss-of-function mechanism rather than a gain-of-function [34, 35]. *PSEN1* and *PSEN2* have important sequence homology also at the protein level [2].

*PSEN1* gene consists of 12 exons that encode a 467-amino acid protein that is predicted to traverse the membrane six to ten times. The amino and carboxyl terminal are both oriented toward the cytoplasm. The majority of *PSEN1* mutations are single-nucleotide substitutions, but small deletions and insertions have been described as well. At present, more than 200 different AD-related mutations have been identified, scattered over the protein with some clustering within the transmembrane domains and the hydrophilic loops surrounding these domains [13, 36, 37].

*PSEN2* has 12 exons and is organized into ten translated exons that encode a 448-amino acid protein. The PSEN2 protein is predicted to consist of nine transmembrane domains and a large loop structure between the sixth and seventh domain and also displays tissue-specific alternative splicing [38]. The mechanism by which *PSEN2 *increases A*β* generation in the brains of AD patients remains to be clarified. A recent study found that mutant *PSEN2* increases *β*-secretase activity through reactive oxygen species-dependent activation of extracellular signal regulated kinase [39].

## 4. Clinical Features of Monogenic AD

In broad terms, the clinical presentation of monogenic AD is similar to that of sporadic AD. However, the phenotype associated with monogenic AD is somewhat broader than what is typically seen in sporadic AD. Moreover, neurologic signs and symptoms appear to be more common in monogenic AD as compared to sporadic forms. The copresence of different elements could help in recognition in clinical practice [37].

### 4.1. Age at Onset and Survival

Overall, monogenic AD usually has an earlier age at disease onset. The youngest age at onset has been described for *PSEN1* mutations; symptoms typically first appear between the age of 30 and 50, but some mutations have been associated with earlier onset [40]. *PSEN1 *mutations show almost complete penetrance by the age of 60, with some exceptions (Table 1). The causes of variability of age at onset are neither clear nor completely explained by genetic factors [41, 42] or by the biochemical abnormalities of A*β* ratio due to the mutations [34]. *APP* pedigrees tend to have an older age at onset, typically in the 50s and ranging from 45 to 60 years old. The rarer *PSEN2* mutations have the widest range of onset with some late-onset cases [43], and the incomplete penetrance has been postulated.

Overall survival in monogenic AD is similar to sporadic disease with an average of 6–9 years from diagnosis, with the caveat that survival in elderly sporadic individuals tends to be lower. The different age at onset does not influence the disease duration, and, in general, *PSEN1 *mutation carriers may have slightly shorter survival.

### 4.2. Cognitive and Behavioural Picture

The majority of monogenic cases have an amnestic presentation very similar to that seen in sporadic disease [44, 45]. Longitudinal studies of unaffected at-risk individuals have suggested that the earliest neuropsychometric findings involve a fall in verbal memory [46, 47], with relatively preserved naming and object perception compared with sporadic AD [44, 45]. However, only few studies conducted a standardised neuropsychological assessment, and atypical cases with subcortical, or aphasic presentation are often reported in the literature [48–50]. Atypical language presentation has been associated with specific *PSEN1* mutations but is relatively rare in *PSEN2* and *APP* cases (see Table 1).

Recent findings also suggest the possibility of “pure” frontotemporal presentation associated with frontotemporal atrophy or hypoperfusion [51, 52]. The presence of epileptic seizure very rare in FTLD spectrum and the CSF AD typical biomarkers may help in the differential diagnosis [51, 53].

Behavioral and psychiatric symptoms (BPSD) such as delusions, hallucinations, and apathy, often present in sporadic AD, could appear also in monogenic cases. In the largest kindred of monogenic AD studied in the *PSEN1*E280A population from Antioquia, memory impairment was detected in 100% of cases, and behavioural changes were present in 94% of individuals [54].

### 4.3. Myoclonus and Seizures

In monogenic AD, the frequency of myoclonus increases with the duration of illness. All monogenic AD forms have been associated with the presence of myoclonus, and some *PSEN1* variations have been linked to the early presentation of this sign. Several reports also suggest myoclonus as a harbinger of the more common seizures.

Seizures could represent the first presentation in many cases of monogenic AD, especially, for *PSEN1* mutations. In clinical practice, in early-onset cases, it could be difficult to differentiate autosomal dominant AD from genetic epilepsy or storage disorder such as neuronal ceroidlipofuscinosis [55]. Some *PSEN1* mutations have been identified in cases with prominent epilepsy at presentation (see Table 1). Seizures are very common in *APP* duplication and Down syndrome that have an extra copy of *APP*, thus reflecting a possible link between A*β* dosage and epilepsy [56]. It has been shown in experimental animals that amyloid *β*-peptides may induce neuronal hyperexcitability and trigger progressive epilepsy [57]. Myoclonus and seizures have not been reported in a few *PSEN1, PSEN2*, and *APP* mutations, but this absence may simply reflects restricted duration of follow-up.

### 4.4. Other Neurological-Associated Signs or Symptoms

*PSEN1* phenotypes also include extrapyramidal, pyramidal, or cerebellar isolated presentation, rarer in *PSEN2* or *APP*-mutated patients (Table 1). However, prominent parkinsonism associated with dementia and visual hallucinations fulfilling diagnostic criteria for Lewy Body Dementia (LBD) have been only rarely associated with *PSEN1 *and *PSEN2* mutations [40, 58, 59].

Spastic paraparesis associated with memory complains has been also associated with certain *PSEN1* mutations [60]. The neuropathological correlate is often the presence of “cotton wool plaques”, consisting of A*β* deposits with a lack of amyloid in the core and poor neuritic and glial response [61].

Cerebellar ataxia or gaze-evoked nystagmus has been only noted occasionally in *PSEN1* mutations carriers. In *PSEN2* and *APP* cases, pyramidal or cerebellar neurological signs could be present but not representing the onset symptom.

### 4.5. Intracerebral Hemorrhages and Cerebral Amyloid Angiopathy

Cerebral amyloid angiopath (CAA) is a generic morphological term describing the pathological changes occurring in cerebral blood vessels resulting from deposition of amyloid proteins of different origins. The most severe clinical consequence of CAA is cerebral haemorrhage, and according to autopsy series, 12 to 25% of all cerebral haemorrhages in the elderly are due to CAA [62, 63].

The first mutation described in the *APP* gene was found within the A*β* region in a family with autosomal form of CAA [64]. In this condition, cerebral haemorrhage was fatal in about two thirds of patients, whilst the one third developed multiple strokes resulting in dementia of vascular type [65]. In 2006, the duplication of *APP* was also associated with a clinical phenotype characterised by a progressive dementia of AD type associated with CAA [17, 30, 31]. Substitution and duplication of *APP* gene have been also associated with variable white matter abnormalities up to severe leukoencephalopathy.

If CAA and cerebral haemorrhage are the key features of *APP* monogenic AD, their presence is only rarely associated with *PSEN1 *or *PSEN2 *phenotypes (Table 1).

## 5. Neuroimaging Features of Monogenic AD

It is well established that in sporadic AD the brain regions early and more severely affected are the medial temporal lobes, especially, the hippocampus and entorhinal cortex, the posterior portion of the cingulate gyrus, and the precuneus [66, 67]. In monogenic AD, several reports showed a similar atrophy pattern with a slight more severe medial-temporal lobe atrophy compared with sporadic AD [68]. Gray matter regional volume loss and decreases in magnetization transfer ratio have also been reported in mildly symptomatic carriers [69]. Additionally, it has been well established that in early onset AD, hippocampus may be not always involved as in the typical form and that frontoparietal areas showed greater atrophy in monogenic forms compared with sporadic late onset cases [68, 70, 71]. *APP *mutations seem to be more associated with hippocampal atrophy, whereas *PSEN1* mutation carriers had more general neocortical involvement and a prominent frontotemporal atrophy [68, 72]. However, given the high heterogeneity of phenotype-genotype correlation in monogenic AD, it would be difficult to find a definitive structural biomarker specific and different for *PSEN1, PSEN2*, or *APP*.

Interestingly, as previously reported, certain mutations within* APP* genes presented leukoencephalopathy that should be evaluated on MRI in order to exclude a possible influence of white matter lesions on cognitive decline [73]. In suspected cerebral amyloid angiopathy, an MRI with gradient echo sequences should be performed to show the presence of cerebral microbleeds (or microhaemorrhages), visualized as small, rounded, dot-like lesions of low signal intensity in the T2*-weighted images [74]. Susceptibility-weighted imaging has considerably increased microbleed detection rates compared with gradient echo sequences [75] although the sensitivity to detect microbleeds is also dependent on slice thickness and magnetic field strength. Microbleeds in deep brain regions are most likely to be associated with vasculopathy owing to hypertension, whilst their distribution is mostly lobar in specific disorders such as sporadic cerebral amyloid angiopathy [76].

In atypical monogenic AD phenotypes, such as epileptic, paraparetic, or ataxic variants, MRI is also essential to distinguish AD from storage or mitochondrial disorders [77], Creutzfeldt-Jacob Disease (CJD) [78], or other specific forms [79].

Along with structural imaging, cerebral blood flow SPECT (single-photon emission computer tomography) and brain FDG-PET (fludeoxyglucose Positron emission tomography) scans in monogenic AD patients show predominant hypoperfusion or reduced glucose metabolism in the temporoparietal regions, including the precuneus and the posterior cingulate cortex, a pattern similar of sporadic AD [80]. However, as outlined in the clinical section, many autosomal dominant cases showed an extended phenotype involving also frontal and prefrontal areas, and cases with pure frontotemporal hypoperfusion pattern have been reported [51, 52].

Only few studies compared the ability of SPECT and FDG-PET to discriminate AD from other dementia. FDG-PET revealed to have higher sensitivity and specificity if compared to SPECT [81, 82]. In atypical focal monogenic AD functional neuroimaging reflects the topographical distribution of neurodegeneration and not the underlying pathology. Thus the role of SPECT and FDG-PET is still controversial.

### 5.1. Amyloid Imaging

More recently, PET amyloid imaging studies with Pittsburgh Compound B (PiB) have revealed evidence of fibrillar A*β* deposition in monogenic AD, including carriers who were up to 10 years younger than the age of onset for their family [83]. Interestingly, these studies have consistently reported elevated levels of PiB retention in the striatum of presymptomatic monogenic AD individuals, which occurs more variably in late-onset sporadic AD [47].

Amyloid imaging such as 11C-PIB PET has very high (90% or greater) sensitivity for AD although the specificity decreases with aging [84]. The amyloid imaging tracers flutemetamol, florbetapir, and florbetaben labelled with 18F demonstrated good accuracy for distinguishing patients with AD from other tauopathies or TDP-43 pathologies [83, 85–87]. Amyloid tracer binding is diffuse and symmetrical, with high uptake consistently found in the prefrontal cortex, precuneus, and posterior cingulate cortex, followed by the lateral parietal, lateral temporalcortex, and striatum.

Another important role of amyloid imaging will be the differential diagnosis of intracranial haemorrhage caused by small vessel disease or cerebral amyloid angiopathy, the last showing positive scans.

## 6. Cerebrospinal Fluid and Blood Biomarkers

In the assessment of a presenile or atypical dementia, cerebrospinal fluid (CSF) should be performed in order to exclude other mimicking diseases. In monogenic AD, multiple groups have reported that CSF A*β*42 is reduced to approximately one-half of normal values [88], a finding remarkably similar to sporadic AD [89]. While decreased A*β*42 appears to have remarkable specificity for pathologic AD and A*β* amyloidosis in the brain [90], CSF A*β*40 is not consistently different in AD individuals compared with normal individuals. However some *PSEN1* mutations have been also associated with increased A*β*42 production reflected also in CSF, thus altering the paradigm of a low A*β*42 in all AD forms [91]. CSF tau and phosphotau levels are increased almost two-fold in monogenic AD individuals compared with controls [88], again mimicking the CSF profile in later onset sporadic AD.

In clinical practice, CSF Tau/A*β*42 ratio may reflect the underlying pathology also in focal atypical presentation such as corticobasal degeneration [92], bvFTD [53] or primary progressive aphasia [93]. As for amyloid imaging, in early-onset cases in which a copathology with AD is very rare, CSF analysis should be performed, and it has a higher specificity value.

Increased plasma A*β*42 has been consistently found in monogenic AD [47, 91], while there is little, if any, consistently reported difference in sporadic AD [94].

The use of new genetic detecting methods, such as next generation sequencing, will probably change the scenario of presenile dementia genetics. A recent screening for 16 different dementia disease genes proposed by Beck et al. [95] at UCL showed a great sensitivity (82%) and specificity (100%) in detecting pathogenic alterations compared with normal methods. Interestingly, *APP* duplication could be missed also with these new approaches, underlying the importance of a right clinical selection of these cases [95].

Gene expression analysis in monogenic forms may also help in the identification of early serum biomarkers [96], as already demonstrated for other forms of dementia [97].

## 7. Presymptomatic at-Risk Subjects

Several series of presymptomatic mutation carriers have been studied in order to elucidate the very early phases of monogenic AD. Owing to the geographically dispersed nature of monogenic AD families and the relative rarity of the disease, an international network of research centres has been established, formally known as the Dominantly Inherited Alzheimer's Network [DIAN]. In 2012, the first DIAN report confirmed that changes begin in the brain about 25 years before expected symptom onset with the decline in A*β*42 concentrations in the CSF in mutation carriers, as compared with noncarriers [47]. A*β*42 deposition as measured by PIB-PET was detected at least 15 years before expected symptom onset [98]. Increases in levels of tau in the CSF and in brain atrophy were detected approximately 15 years before expected symptom onset, followed by cerebral hypometabolism and impaired episodic memory approximately 10 years before expected symptom onset and global cognitive impairment starting at 5 years before expected symptom onset.

Longitudinal structural imaging studies have demonstrated alterations in white matter structure in presymptomatic and early symptomatic carriers, with decreased fractional anisotropy in the fornix and widespread areas of brain visualized with diffusion tensor imaging [99]. Several neuroimaging studies showed that even before the bilateral hippocampal atrophy, presymptomatic mutation carriers have an increase caudate size [100] and early thalamus involvement [101]. The grey matter atrophy may be not restricted to hippocampus but also to other cortical areas, especially, precuneus, parietal, or frontal brain regions [71, 102]. A recent DIAN study on more than 100 presymptomatic and symptomatic carriers confirmed the early thalamus involvement and showed white matter atrophy in the cingulum and fornix [103]. Functional connectivity has also recently showed the early disruption of the default mode network in monogenic AD even before the symptoms presentations [104].

## 8. Conclusions

The wide spectrum of presentation of monogenic AD leads often to late diagnosis or missidentification of cases.

The memory impairment, still essential for the new revised research criteria in association with CSF or imaging biomarkers for the diagnosis of AD [105], is not always the prominent early deficit. Behavioural disturbances, epilepsy, myoclonus, or CAA (specific for *APP* mutations) may help in addressing diagnosis. However, structural or functional neuroimaging is more consistent with focal phenotypes than the AD pathology. Thus, CSF or amyloid imaging may be useful in the differential diagnosis with other neurodegenerative dementias, especially in early onset cases, but these biomarkers cannot be considered specific for the different involved genes.

In cases suggestive for autosomal dominant AD, we suggested a screening of *PSEN1* mutation first, followed by *APP* and *PSEN2* mutations on the basis of epidemiological data, but new specific biomarkers driving genetic screening are warranted. Since more than 20 years, the study of familial forms is giving a big contribution in the understanding of the underpinning mechanisms of AD and possible target approaches. Thus, a very early identification of monogenic cases is pivotal in the development and evaluation of disease-modifying therapy needed also in the most common sporadic form.

## Figures and Tables

**Table 1:** Atypical presentation of different *PSEN1*, *PSEN2*, and *APP* mutations.

Clinical phenotype	Mutations		Differential diagnosis
Very early onset (<30 y)	*PSEN1*	L85P P117L P117S L166P S169L M233L M233V L235P Y256S V272A A434C P436Q G206V	GE, MD, SD, PWMD, HD

Late onset (>65 y)	*PSEN1 *	Uncommon, A79V M139V I143F H163R H163Y A231V K239N L271V E273A R377W C410Y	Sporadic AD, FTD, LBD, VaD, CJD
	*PSEN2 *	Possible for all mutations, especially, M239I and M239V	

Behavioral or psychiatric symptoms	*PSEN1 *	Common at presentation V89L C92S P117L M139V M146I M146L H163R H163Y L166P L166R S169L F176L E184D I202F G206A G206V K239N L250S L250V Y256S R269G R269H V272A E280A L282V*Δ*9 R377W L392P C410Y L424P A434C	bvFTD, LBD, HD, WE, CJD
	*PSEN2 *	Possible at presentation R71W A85V T122P T122R Y231C M239V M239I	
	*APP *	Rare at presentation D694N A713T	
“Pure” frontotemporal presentation	*PSEN1 *	L113P P117R M139V M146V L174R G183V L226F M233L M233T P264V E280A V412I L424H	

Prominent aphasia	*PSEN1 *	E120D H163R H163Y L166R G209V L226F L235R A246E L250S A260V L262F P264L R278I E280A R377W L392V A431E A434C L435F	Sporadic PPA (svPPA, lvPPA, avPPA) FTD-MND, CJD
	*PSEN2 *	Y231C	

Epileptic seizures	*PSEN1 *	Possible as first presentation; L113P L113Q intron4insTAC P117L E120D E120G N135S M139I M139V I143T M146I M146L H163P H163R H163Y L166R S169L S169P S170F E184D G206D G206V Q222H M233T M233V F237I A246E L250V A260V P264L R269G R269H E280A E280G L282R L282V L282VΔ9 R377W L392V L420R L424P A434C	GE, SD, MD, AE, CJD
	*PSEN2 *	Rare at presentation, often seen during disease course M239V	
	*APP *	Possible at presentation for D694N *APP* duplication (related or not to ICH)	

Myoclonus	*PSEN1 *	L113P 4insTAC P117R M139V I143T M146L M146I M146V L153V H163R H163Y S169P S169L L174M E184D G209V Q222H F237I L250V L250S A260V P264L R269H R269G E280A L286V L392V C410Y A434C	CBS, MD, GE, CJD

Parkinsonism, dystonia, or apraxia	*PSEN1 *	C92S F105L L113P P117R E120D N135D M139V M146I M146L M146V H163P H163R H163Y L166R S170F E184D I202F G206A G209V G217D M233L M233T M233V F237I L250S Y256S G266S V272A R278I E280A P284L L286VΔ9 L381V L392V C410Y A431E L435F ΔT440	CBS, LBD, PSP, FTD, CJD
	*PSEN2 *	Rare at presentation, possible during disease course A85V M239V	

Spastic paraparesis	*PSEN1 *	ΔI83/M84, L85P N135S Y154N InsF1 L166P G217D F237I V261F V261L P264L P264V G266S L271V R278K R278S R278T E280G E280Q P284L P284S L286R Δ9 Ins18 T291P L381V, N405S L424R P436Q	HSP, VaD, PWMD, MND, CJD

Cerebellar ataxia	*PSEN1 *	P117A N135S M139V I143T H163P L166P S169L S170F Y256S E280A L282V P436Q	CJD, SCA, MSA-C, PNS

Leukoencephalopathy	*APP *	D694N A713T	VaD, PWMD, DD, V

CAA with or without ICH	*PSEN1 *	Rare ΔE4 V89L 4insTAC E184D C217D L271V V272A E280G L282V S290C N405S ΔT440	Sporadic CAA, monogenic CAA (*CYST C, TTR, ITM2B, PRNP* mutations)
	*PSEN2 *	Rare R71V N141I	
	*APP*	Often present also without cognitive impairment A692G E693Q E693G E693K D694N A713T *APP* duplication	

Abbreviations: AD: Alzheimer's Disease, AE: autoimmune encephalitis, *APP*: Amyloid Precursor Protein gene, avPPA: agrammatic variant of primary progressive aphasia, bvFTD: behavioural variant of frontotemporal dementia, CAA: cerebral amyloid angiopathy, CBS: corticobasal syndrome, CJD: Creutzfeldt-Jacob Disease, *CYST C*: Cystatin C gene, DD: demyelinating Disease, FTD: frontotemporal dementia, GE: genetic epilepsy HD: Huntington's Disease, HSP: hereditary spastic paraplegia, *ITM2B*: integral membrane protein 2B gene, LBD: Lewy Bodies Dementia, lvPPA: logopenic variant of primary progressive aphasia, MD: Mitochondrial Disease, MND: Motor-neuron Disease, MSA-C: cerebellar form of multisystem atrophy, PNS: paraneoplastic syndromes, *PRNP*: prion protein gene, *PSEN1*: Presenilin 1 gene, *PSEN2*: Presenilin 2 gene, PSP: progressive supranuclear palsy, PWMD: progressive white matter disease, SCA: spino cerebellar ataxia, SD: storage disorders, svPPA: semantic variant of primary progressive Aphasia, *TTR*: Transthyretin gene, VaD: Vascular dementia, V: Vasculitis, WE: Wernicke encephalopathy.
